# Psychosocial Determinants of Fruit and Vegetable Intake in Japanese Adolescents: A School-Based Study in Japan

**DOI:** 10.3390/ijerph17155550

**Published:** 2020-07-31

**Authors:** Yoshiko Sato, Masamitsu Miyanaga, Da-Hong Wang

**Affiliations:** 1 Department of Biochemistry, Okayama University of Science, Okayama 700-0005, Japan; yo-sato@a.zaq.jp (Y.S.); miyanaga@dbc.ous.ac.jp (M.M.); 2 Science Division, Wakayama Shin-ai Junior and Senior High School, Wakayama City 640-8151, Japan

**Keywords:** vegetable and fruit, psychosocial determinants, social support, perceived barrier, adolescents

## Abstract

A few studies in Japan have demonstrated positive attitudes, self-efficacy, social support, and perceived barrier were associated with fruit and vegetable (F&V) intake in adults; however, limited evidence addresses the association of psychosocial factors with F&V intake in adolescents. A cross-sectional study through a questionnaire survey was conducted at junior and senior high schools, and 933 students completed the questionnaire. Data were analyzed by X^2^ test and Student *t*-test. The findings demonstrated 2.7% of participants were aware of the current recommendations for vegetable and 2.0% for fruit. Only 4% and 8.1% of participants reported they consumed recommended amount of vegetables and fruits. In comparison with males, females showed higher scores of attitude (*p* < 0.01), responsibility (*p* < 0.01), and social support (*p* < 0.01). The barriers to vegetable intake were “I’m eating enough now”, “not always available when eating away from home”; the barriers to fruit intake were “don’t have a habit of having 100% juice or fruit in the morning”, and “cost too much”. The findings suggest the change of adolescents’ knowledge about what they should eat is needed in boosting F&V consumption. The development of an intervention program for adolescents needs to target socio-environmental factors such as family support, and nutritional education for early healthy habit formation.

## 1. Introduction

Many studies have suggested that fruit and vegetable (F&V) intake decreases the risk of coronary heart disease, cancer, type 2 diabetes, hypertension, and stroke through beneficial influences of possessing such as counteraction of inflammatory processes, decrease in platelet aggregation, alterations in the cholesterol metabolism, modulation of detoxification enzymes, and stimulation of the immune system [1,2,3,4,5,6]. In 2000, the Health Japan 21, a nationwide campaign for prevention of lifestyle-related diseases, was launched, and the 2nd term of the campaign started from 2013. The daily recommended amounts of fruit and vegetables are 200 g or more and 350 g or more, respectively, by Health Japan 21 (from the Ministry of Health, Labor and Welfare) and the guidelines for a balanced diet (from the Ministry of Agriculture, Forestry and Fisheries) [7,8]. However, for people aged 15–19, recent Japan’s National Health and Nutrition Survey (NHNS) in 2018 showed the average consumption of fruit was 62.1 g and of vegetables was 256.7 g per day [9]. It is known that the nutrient needs of adolescents are greater because of accelerated physical growth rate during this period [10]. Park et al. reported that a high intake of F&V was associated with better perceived general health, oral health, happiness, and sleep satisfaction among Korean adolescents [11]. However, younger children are more likely to consume F&V than adolescents, and a reduction in F&V consumption was observed among adolescents in UK and USA [12,13]. Data based upon the Global School-Based Health Survey from five Southeast Asian countries (India, Indonesia, Myanmar, Sri Lanka, and Thailand) showed over 76.3% in-school adolescents (13–15 years of age) consumed fewer than five servings of F&V per day [14]. Therefore, understanding the real reasons why adolescents do not consume more F&V is crucial to the development of effective intervention programs.

It is considered that dietary behavior established in childhood or adolescence is possibly carried over into adulthood [15], therefore understanding the health benefit of F&V consumption as early as possible might contribute to the slowing down of the onset of chronic diseases in their adulthood. Many studies demonstrated that psycho-social factors are related to F&V consumption [16,17,18,19]. A review of 35 studies suggested that self-efficacy, social support, and knowledge were strong predictors of adult F&V consumption [17]. Studies in India found unhealthy school food environments/home food environments, lack of knowledge regarding nutrition and healthy meal preparation among parents and school canteen staff, inappropriate school food policy, etc., were critical barriers to the promotion of adolescents’ healthy eating [20,21,22]. Some studies in Japan have demonstrated that positive attitudes, self-efficacy, social support, and perceived barriers were associated with daily F&V consumption in adults [23,24,25,26,27]. However, there is limited evidence that addresses the association of psychosocial factors (psychological and social aspects such as thought, feeling, attitudes, social environment, etc., that affect individuals’ behavior [28]) with daily F&V intake among adolescent population. To facilitate future effective intervention programs for increasing F&V intake, we investigated psychosocial factors related to F&V consumption among adolescents in Japan. 

## 2. Subjects and Methods

### 2.1. Study Design and Participants 

A cross-sectional study was conducted at two junior and senior high schools in Okayama city and Wakayama city, Japan. We recruited the subjects from June 2018 to February 2020, and 933 (male: 332, female: 601) students aged 12–18 years agreed to participate in the study. A self-administered questionnaire survey was carried out for participants’ demographic characteristics (Table 1), self-reported daily F&V intake (instructions and example were provided in the questionnaire), knowledge of target F&V level, and psychosocial parameters related to F&V intake as described previously [18]. The original questionnaire has been used in successfully implementing and evaluating interventions in community-based settings to increase F&V intake in WIC (Women, Infant, and Children) 5-A-Day promotion program [16]. It was translated into Japanese, and then modified and tested in a pilot study for use in the adult Japanese population and adolescents as well [27]. The study design was approved by the Ethics Committee of Okayama University of Science (No. 30-3). The study was carried out in accordance with the Declaration of Helsinki, the study objective was explained to all participants, and verbal informed consent was obtained from them prior to enrollment in the questionnaire survey. 

### 2.2. Psychosocial Parameters

We examined psychosocial parameters (nutritional knowledge, attitudes, responsibility, self-efficacy, social support, and perceived barriers) by the questionnaire (Table 2). Nutritional knowledge of the participants regarding F&V intake was measured with five questions. Attitudes toward F&V intake were measured with five questions, and each answer was scored on a five-point Likert scale ranging from “agree a lot” to “disagree a lot”. Responsibility for food preparation (three questions) and self-efficacy for eating F&V (eight questions) were assessed by three-point Likert scale (“Sure”, Somewhat Sure”, “Unsure”). Social support was evaluated with three items by asking “Are there other people encouraging you to (1) buy F&V; (2) prepare F&V; and (3) eat more F&V. Perceived barriers were measured with 18 items (Table 3 and Table 4), and each item was scored on a five-point Likert scale ranging from “agree a lot” to “disagree a lot”. Further details on psychosocial parameters are described elsewhere [27]. 

We also asked the participants if they knew how much F&V they should eat per day; if the answer was “Yes”, they were further required to write down the amount (grams) of F&V they should consume in a day as recommended by Health Japan 21 and the Guidelines for a balanced diet [7,8]. 

### 2.3. Body Mass Index

Body mass index (BMI) of each participant was obtained from self-reported height and weight, and it was calculated as weight divided by height squared (kg/m^2^). Participants with BMI ≥ 25 were considered overweight, <18.5 were categorized as underweight, and those within 18.5–24.9 were considered normal weight. 

### 2.4. Data Analysis

Two-group comparisons were performed using Student *t*-test for the continuous variables and Pearson’s Χ^2^ test for the categorical variables. The relationship between daily F&V consumption and psychosocial factors was analyzed by Spearman’s rank correlation analysis.

All statistical analyses were performed using SPSS Statistics Version 26.0 for Windows (SPSS Inc., Chicago, IL, USA).

## 3. Results

Table 1 demonstrates the characteristics of the participants. No significant differences were observed between male and female for age and BMI. About 9.1% of the participants reported “Skipping breakfast” more than 3 days a week; one-third of participants reported “Eating something within two hours before bedtime” more than 3 days a week. The proportion of males who had sports habits was significantly higher than those of females (72.3% vs 48.9%, *p* < 0.001). 

Only 2.7% of participants were aware of the current recommendations for vegetable and 2.0% for fruit. The majority of the participants, males in particular, stated that they did not know their daily vegetable (male 72.9% vs. female 61.9%, *p* = 0.009) and fruit (male 69.0% vs. female 54.7%, *p* = 0.002) consumption. The average amount of vegetable consumption was 171.3 g, and fruit consumption was 89.9 g (Table S1). Only 4% and 8.1% of participants reported that they consumed recommended daily amount of vegetables (≥350 g) and fruits (≥200 g), respectively. Of the 933 participants, about 20% of them consumed less than 200 g of vegetables (male 16.0% vs. female 23.5%, *p* = 0.009) and less than 100 g of fruits (male 18.7%, female 20.6%) per day.

Table 2 shows the differences in psychosocial profiles related to F&V consumption by gender. In comparison with males, females showed higher scores of nutritional knowledge (*p* = 0.068), attitude (the importance of F&V consumption) (*p* < 0.01), responsibility (food purchasing and preparing/meal planning) (*p* < 0.01), and social support (the existence of others who encourage F&V consumption) (*p* < 0.01).

The main perceived barriers to vegetable consumption were “I think I’m eating enough now” (mean scores: male 3.06 ± 0.08 vs. female 2.99 ± 0.05, *p* = 0.417) “Vegetables are not always available when I eat away from home” (mean scores: male 2.63 ± 0.08 vs. female 2.81 ± 0.05, *p* = 0.05) and “Vegetables cost too much” (mean scores: male 2.64 ± 0.08 vs. female 2.76 ± 0.06, *p* = 0.153) (Table 3). The main perceived barriers to fruit consumption were “Having 100% juice or fruit in the morning is difficult because I don’t have such a habit” (mean scores: male 3.07 ± 0.09 vs. female 3.11 ± 0.06, *p* = 0.751) “Fruits cost too much” (mean scores: male 2.68 ± 0.08 vs. female 2.92 ± 0.06, *p* = 0.015), and “Fruits are not always available when I eat away from home” (mean scores: male 2.58 ± 0.08 vs. female 2.85 ± 0.06, *p* = 0.005) (Table 4).

In order to examine whether there is any difference in F&V consumption between the highest or the lowest group of BMI and the standard group, we divided the participants into three groups based on their BMI values (BMI < 18.5, BMI: 18.5–24.9, BMI ≥ 25). We found those who reported the lowest consumption of F&V were in the highest BMI group (BMI ≥ 25) although statistically non-significant (Table 5).

The results on relation between daily amount of F&V consumption and scores of psychosocial determinants indicated that the scores of attitude and self-efficacy positively correlated with daily vegetable consumption (Table 6), and the scores of attitude, responsibility, social support, and self-efficacy positively correlated with daily fruit consumption (Table 7). However, the scores of perceived barriers inversely correlated with perceived barriers to both vegetable and fruit consumption (Table 6 and Table 7). 

## 4. Discussion

So far there is a scarcity of information about public awareness of the recommendations for F&V in Japan. Recent research for people over 18 years old demonstrated less than one-fourth of the participants were aware of this recommendation [27]. The current finding in adolescents showed that less than 3% knew that they should eat at least 350 g of vegetables and 200 g of fruits in a day, indicating the apparent lack of public awareness of the recommendations might be one of the main reasons for low level of daily F&V consumption. A lot of works have shown the “5-a-Day for Better Health Campaign” that started in 1991 in the USA was successful in increasing the public awareness of the recommendations for daily F&V intake by “its use of mass media, its partnership between the state health department and the produce and supermarket industries” [29,30] and as well as in increasing the consumption of F&V through worksite approaches, family-based interventions, direct mail intervention, etc. [16,30,31]. In addition, many countries have also shown increased consumption of F&V among children and adolescents by such as school-based education and gardening-based interventions [32,33,34,35,36,37,38,39]. 

The current finding also indicated that most participants did not know how much vegetables or fruits they were eating in a day, particularly in male adolescents (for vegetables: males: 72.9%, females: 61.9%; for fruits: males: 69.0%, females: 54.7%). For those who knew daily amount of F&V consumption, most of them ate much less than the recommended amounts of vegetables and fruit in both male and female; only a small proportion of participants met the target level (4% for vegetables, 8.1% for fruits). In line with an Indian research by Rathi et al. [40], our findings also showed female adolescents consumed more F&V than males. In both male and female adolescents, the highest score of perceived barriers to vegetable consumption was “I think I’m eating enough now”, and to fruit consumption was “Having 100% juice or fruit in the morning is difficult because I don’t have such a habit”. Data based on the Western Australian component of the National Secondary Students’ Diet and Activity survey (2009–2010 and 2012–2013) demonstrated that half of the adolescents (aged 12–17 years) who were not consuming the recommended servings of F&V did not perceive their intake to be inadequate [41]; our finding also demonstrated most participants who were not consuming the recommended amount of F&V perceived their intake to be “enough”. Therefore, strategies for broad recognition of target level for fruit and vegetable intake and its health benefits are definitely necessary to increase the adolescents’ consumption of F&V.

In 2000, when the Health Japan started, the NHNS demonstrated the average daily intake of vegetables and fruits was 236.1 g and 98.5 g (15–19 years old) [42]; however, after 18 years the results of 2018 survey [9] still did not show marked improvement (256.7 g, 62.1 g, respectively), and the fruit intake even decreased, implying this nationwide campaign did not successfully boost consumption of vegetables and fruits. Our current finding (by self-reported, 12–18 years old) also showed low vegetable (171.3 ± 7.2 g) and fruit (89.9 ± 5.3 g) consumption (Table S1), in which daily average amount of vegetable consumption was lower and fruit consumption was higher than the results of the NHNS in 2018 [9]. Such a difference may be partially due to the difference of food record method and the regions the participants from (Okayama city and Wakayama city). These two regions are famous for their fruit product in Japan. The participants from these regions may consume more fruits than people in other regions. 

In terms of psychosocial factors regarding the consumption of F&V, we found female adolescents had significantly higher scores of nutritional knowledge, attitude, responsibility, and social support when compared to male adolescents, and males tended to have higher perceived barrier to F&V consumption. These results were consistent with that in people over 18 years old [8]. The reasons why eating more F&V is difficult for male adolescents were “I don’t like F&V”, “My family don’t like F&V”, and “I’m eating enough now”. For female adolescents, “F&V are not always available when I eat away from home”,” Few kinds of vegetables are available in the winter”, and “Fruits cost too much” showed higher scores. In addition, the average score of social support in female adolescents was 1.4-fold higher than in males, indicating the stronger social support provided by family, friends, etc., may strongly affect the adolescents’ choice to diet [31,43]. A study in nine private secondary schools of urban India also found that over 60% of students (aged 14–16 years) thought their current F&V intakes are adequate [44], although previous research showed inadequate number of F&V intakes among many of these students [40]. Recent studies showed that parental support was associated with higher F&V consumption among Texas 8th- and 11th-grade students in USA [45] and 10th- and 12th-grade students in Dubai [46]. Unhealthy school food environments/home food environments, lack of knowledge regarding nutrition and healthy meal preparation among parents and school canteen staff, and inappropriate school food policy affect adolescents’ food choice and eating behaviors [20,21,22]. In addition, our findings also indicated self-efficacy positively correlated with F&V consumption, especially in female adolescents, which is consistent with the results by Luban et al. [47]. 

It is worthy of note that those who reported lower consumption of F&V were in the group of higher BMI (BMI ≥ 25). Several studies for both adolescents and adults also indicated that overweight people ate fewer fruits or vegetables [14,19,48]. Since there were only seven people in the overweight group (Table 5), and data on total energy intake and expenditure were not available, further study is needed to confirm the relation between F&V intake and BMI. 

We found 9.1% of the participants reported “Skipping breakfast” more than 3 days a week, and the proportion of breakfast skippers (once or more per week) was higher in female (18.1%) than in males (15.6%), which is consistent with the study in UK [49]. A study in urban Baroda (India) demonstrated that 16.1% of adolescents had their breakfast only once or twice a week and 12.6% never had breakfast [50]. Matsumoto et al. recently reported that breakfast skipping was related to deficiencies in vitamin and mineral intakes among Japanese female junior high school students [51]. The research by Lim et al. demonstrated that about 40% of female adolescents who skipped breakfast daily had irregular menstruation in South Korea [52]. 16% had their breakfast only once or twice a week, and 12 % of adolescents never had breakfast.

The current study is one of the few examining the psychosocial determinants of F&V consumption among teenagers in Japan. The significance of the current findings is that it raised basic issues that need to be solved for the promotion of F&V consumption in Japanese adolescents, and it would be a useful reference for designing effective intervention programs. There are several potential limitations to this study. First, this is a cross-sectional study, which cannot determine the casual relationship between psychosocial factors and F&V. Second, the current findings are based upon the participants’ self-report; they might be vulnerable to reporting bias. Third, the recruitment process was conventional and the data were recruited from only two schools in Japan; the results should be interpreted with caution. Finally, Roos et al. [53] reported people with higher socioeconomic status consumed more F&V than people with lower socioeconomic status. However, information on household income was unavailable in this study. 

## 5. Conclusions

A majority of adolescent participants did not know the target level for F&V, suggesting the change of adolescents’ knowledge about what they should eat is needed for success in boosting F&V consumption. Future intervention programs to increase F&V consumption in adolescents need to target socio-environmental factors such as family support, nutritional education in school for early healthy habit formation, and increased availability of F&V with low cost. 

## Figures and Tables

**Table 1:** Participants Characteristics.

Characteristic			Total (*n* = 933)	Male (*n* = 332)	Female (*n* = 601)
Age (mean ± SD)			15.7 ± 1.0	15.8 ± 0.7	15.7 ± 1.2
BMI (mean ± SD)			19.9 ± 2.3	19.7 ± 2.2	20.0 ± 2.4
Awareness of the recommended amount of daily vegetable intake					
	Yes		25 (2.7)	11 (3.3)	14 (2.3)
	No		908 (97.3)	321 (96.7)	587 (97.7)
Awareness of the recommended amount of daily fruit intake					
	Yes		19 (2.0)	9 (2.7)	10 (1.7)
	No		914 (98.0)	323 (97.3)	591 (98.3)
Self-reported daily vegetable intake (g/day)					
	<200		194 (20.8)	53 (16.0)	141 (23.5) **
	200–349		88 (9.4)	26 (7.8)	62 (10.3)
	≥350		37 (4.0)	11 (3.3)	26 (4.3)
	I don’t know		614 (65.8)	242 (72.9)	372 (61.9) **
Self-reported daily fruit intake (g/day)					
	<100		186 (19.9)	62 (18.7)	124 (20.6)
	100–199		95 (10.2)	24 (7.2)	71 (11.8) **
	≥200		76 (8.1)	17 (5.1)	59 (9.8) **
	I don’t know		576 (61.7)	229 (69.0)	347 (54.7) **
Skipping breakfast					
	Almost every day		52 (5.6)	22 (6.6)	30 (5.0)
	3 to 4 days a week		33 (3.5)	15 (4.5)	18 (3.0)
	1–2 days a week		76 (8.1)	15 (4.5)	61 (10.1) *
	rare		762 (81.7)	276 (83.1)	486 (80.9)
	Non-response		10 (1.1)	4 (1.2)	6 (1.0)
Eating and drinking within 2 h of bedtime					
	Almost every day		145 (15.5)	64 (19.3)	81 (13.5) **
	3 to 4 days a week		154 (16.5)	70 (21.1)	84 (14.0) **
	1–2 days a week		229 (24.5)	66 (19.9)	163 (27.1) **
	rare		394 (1.8)	127 (38.3)	267 (44.4)
	Non-response		11 (1.2)	5 (1.5)	6 (1.0)
Sports habit					
	Yes		534 (57.2)	240 (72.3)	294 (48.9) **
	No		387 (41.5)	88 (26.5)	299 (49.8) **
	Non-response		12 (1.3)	4 (1.2)	8 (1.3)

Values in parentheses denote percentages; * *p* < 0.05; ** *p* ≤ 0.01; (Male vs. female by Chi-square test).

**Table 2:** Mean scores of psycho-social determinants for fruit and vegetable intakes by gender.

Parameter	Total	Male	Female
Knowledge	2.64 ± 0.03 (911)	2.55 ± 0.06 (320)	2.68 ± 0.04 (591)
Attitude	19.4 ± 0.16 (923)	18.5 ± 0.30 (327)	19.9 ± 0.18 (596) **
Responsibility	4.13 ± 0.04 (926)	3.87 ± 0.06 (329)	4.27 ± 0.05 (597) **
Social support	1.23 ± 0.04 (921)	0.97 ± 0.06 (327)	1.38 ± 0.05 (594) **
Self-efficacy	17.1 ± 0.14 (923)	17.2 ± 0.25 (328)	17.1 ± 0.17 (595)
Perceived barriers	42.9 ± 0.42 (881)	43.0 ± 0.81 (306)	42.8 ± 0.47 (575)

Values are mean ± standard error of the mean. Values in parentheses denote the number of respondents; ** *p* < 0.01 (Male vs. female by Student *t* Test). Data were log-transformed before the analysis.

**Table 3:** Mean scores of perceived barriers to vegetable intake.

Perceived Barriers	Total	Male	Female
Eating more vegetables is difficult because			
I don’t like them	2.33 ± 0.05	2.39 ± 0.08	2.30 ± 0.06
they are not always available when I eat away from home	2.75 ± 0.04	2.63 ± 0.08	2.81 ± 0.05 *
my family don’t like them	1.60 ± 0.03	1.76 ± 0.06	1.51 ± 0.04 **
I don’t know how to cook vegetables/cooking is troublesome	2.00 ± 0.04	2.01 ± 0.07	1.99 ± 0.05
I’m eating enough now	3.01 ± 0.04	3.06 ± 0.08	2.99 ± 0.05
Eating 5 servings of vegetables a day is difficult because			
they cost too much	2.72 ± 0.04	2.64 ± 0.08	2.76 ± 0.06
we run out of them at home	2.67 ± 0.04	2.65 ± 0.07	2.68 ± 0.05
few kinds are available in the winter	2.46 ± 0.04	2.34 ± 0.07	2.53 ± 0.05 *

Values are mean ± standard error of the mean.* *p* < 0.05; ** *p* < 0.01 (Male vs. female by Student *t*-test). Data were log-transformed before the analysis.

**Table 4:** Mean scores of perceived barriers to fruit intake.

Perceived Barriers	Total	Male	Female
Eating more fruits is difficult because			
I don’t like them	1.54 ± 0.04	1.69 ± 0.07	1.46 ± 0.04 **
they are not always available when I eat away from home	2.76 ± 0.05	2.58 ± 0.08	2.85 ± 0.06 **
my family don’t like them	1.49 ± 0.03	1.63 ± 0.06	1.42 ± 0.03 **
it is troublesome to eat fruits	2.04 ± 0.04	2.15 ± 0.08	1.97 ± 0.05
I’m eating enough now	2.60 ± 0.05	2.75 ± 0.08	2.51 ± 0.05 *
Eating 200 g of fruits a day is difficult because			
they cost too much	2.83 ± 0.05	2.68 ± 0.08	2.92 ± 0.06 *
we run out of them at home	2.65 ± 0.05	2.64 ± 0.08	2.65 ± 0.06
few kinds are available in the winter	2.27 ± 0.04	2.25 ± 0.07	2.29 ± 0.05
Having 100% juice or fruit in the morning is difficult because			
they are not filling	2.08 ± 0.04	2.09 ± 0.07	2.08 ± 0.05
I don’t have such a habit	3.10 ± 0.05	3.07 ± 0.09	3.11 ± 0.06

Values are mean ± standard error of the mean. * *p* < 0.05; ** *p* < 0.01 (Male vs. female by Student *t*-test). Data were log-transformed before the analysis.

**Table 5:** Daily consumption of fruits and vegetables by body mass index (BMI) category.

Parameter	BMI < 18.5	BMI 18.5–24.9	BMI ≥ 25
Fruits (g/day)	96.3 ± 11.8 (98)	90.5 ± 6.08 (233)	77.9 ± 29.1 (7)
Vegetables (g/day)	174.1 ± 14.1 (88)	176.1 ± 8.91 (210)	119.3 ± 42.0 (7)

Values are mean ± standard error of the mean. Statistically non-significant in comparison with the group of BMI 18.5–24.9 by Student *t*-test. Data were log-transformed before the analysis.

**Table 6:** Correlation between daily vegetable consumption and psychosocial determinants.

Parameter	Total		Male		Female	
	*r*	*p*	*r*	*p*	*r*	*p*
Knowledge	0.072 (314)	0.203	−0.028 (88)	0.795	0.114 (226)	0.087
Attitude	0.149 (316)	0.008	0.087 (89)	0.419	0.181 (227)	0.006
Responsibility	0.079 (317)	0.161	0.049 (89)	0.646	0.086 (228)	0.198
Social support	0.023 (317)	0.685	−0.173 (90)	0.102	0.104 (227)	0.119
Self-efficacy	0.373 (316)	<0.001	0.330 (88)	0.002	0.400 (228)	<0.001
Perceived barriers	−0.179 (300)	0.002	−0.109 (82)	0.332	−0.210 (218)	0.002

Values in parentheses denote the number of respondents. The *r* denotes correlation coefficient by Spearman’s rank correlation analysis.

**Table 7:** Correlation between daily fruit consumption and psychosocial determinants.

Parameter	Total		Male		Female	
	*r*	*p*	*r*	*p*	*r*	*p*
Knowledge	0.012 (352)	0.825	−0.010 (101)	0.921	0.020 (251)	0.753
Attitude	0.177 (355)	0.001	0.167 (103)	0.091	0.187 (252)	0.003
Responsibility	0.135 (355)	0.011	0.199 (102)	0.045	0.102 (253)	0.105
Social support	0.196 (355)	<0.001	0.038 (103)	0.704	0.239 (252)	<0.001
Self-efficacy	0.309 (352)	<0.001	0.258 (100)	0.009	0.319 (252)	<0.001
Perceived barriers	−0.345 (338)	<0.001	−0.337 (94)	0.001	−0.349 (244)	<0.001

Values in parentheses denote the number of respondents. The *r* denotes correlation coefficient by Spearman’s rank correlation analysis.

## Supplementary Materials

The following are available online at https://www.mdpi.com/1660-4601/17/15/5550/s1, Table S1: Daily intake of fruits and vegetables by gender.
