# Supplementary material for: Psychosocial Determinants of Fruit and Vegetable Intake in Japanese Adolescents: A School-Based Study in Japan
Source: Int J Environ Res Public Health. 2020 Jul 31;17(15):5550. doi: 10.3390/ijerph17155550 (PMC7432351; doi:10.3390/ijerph17155550)
Supplement: Supplementary file 1 [file ijerph-17-05550-s001.pdf]

Table S1. Daily intake of fruits and vegetables by gender.

	**Total**	**Male**	**Female**
Fruits (g/day)	89.9 ± 5.31 (357)	79.4 ± 10.2 (103)	94.1 ± 6.20 (254)
Vegetables (g/day)	171.3 ± 7.17 (319)	178.7 ± 16.0 (90)	168.3 ± 7.77 (229)

Values are mean ± standard error of the mean. Values in parentheses denote the number of respondents.
